# The Two-Faced Role of SIRT6 in Cancer

**DOI:** 10.3390/cancers13051156

**Published:** 2021-03-08

**Authors:** Francesco Fiorentino, Vincenzo Carafa, Gregorio Favale, Lucia Altucci, Antonello Mai, Dante Rotili

**Affiliations:** 1 Department of Chemistry, University of Oxford, South Parks Road, Oxford OX1 3QZ, UK; francesco.fiorentino@chem.ox.ac.uk; 2 Department of Precision Medicine, Università degli Studi della Campania “L. Vanvitelli”, 80138 Naples, Italy; vincenzo.carafa@unicampania.it (V.C.); gregorio.favale@unicampania.it (G.F.); 3 Department of Drug Chemistry & Technologies, Sapienza University of Rome, P. le A Moro 5, 00185 Rome, Italy

**Keywords:** NAD^+^-dependent deacylases, cell death modulation, SIRT6 modulators, cancer, epigenetics

## Abstract

**Simple Summary:**

Cancer therapy relies on the employment of different strategies aimed at inducing cancer cell death through different mechanisms, including DNA damage and apoptosis induction. One of the key regulators of these pathways is the epigenetic enzyme SIRT6, which has been shown to have a dichotomous function in cell fate determination and, consequently, cancer initiation and progression. In this review, we aim to summarize the current knowledge on the role of SIRT6 in cancer. We show that it can act as both tumor suppressor and promoter, even in the same cancer type, depending on the biological context. We then describe the most promising modulators of SIRT6 which, through enzyme activation or inhibition, may impair tumor growth. These molecules can also be used for the elucidation of SIRT6 function, thereby advancing the current knowledge on this crucial protein.

**Abstract:**

Sirtuin 6 (SIRT6) is a NAD^+^-dependent nuclear deacylase and mono-ADP-ribosylase with a wide spectrum of substrates. Through its pleiotropic activities, SIRT6 modulates either directly or indirectly key processes linked to cell fate determination and oncogenesis such as DNA damage repair, metabolic homeostasis, and apoptosis. SIRT6 regulates the expression and activity of both pro-apoptotic (e.g., Bax) and anti-apoptotic factors (e.g., Bcl-2, survivin) in a context-depending manner. Mounting evidence points towards a double-faced involvement of SIRT6 in tumor onset and progression since the block or induction of apoptosis lead to opposite outcomes in cancer. Here, we discuss the features and roles of SIRT6 in the regulation of cell death and cancer, also focusing on recently discovered small molecule modulators that can be used as chemical probes to shed further light on SIRT6 cancer biology and proposed as potential new generation anticancer therapeutics.

## 1. Introduction

Sirtuin 6 (SIRT6) is a crucial chromatin regulating protein belonging to the Sirtuin (SIRT) family, a class of broad-spectrum protein deacylases that utilize NAD^+^ as co-substrate [1]. Sirtuins have been initially classified as class III histone deacetylases (HDACs), indeed SIRT6 has been shown to catalyze the deacetylation of lysines K9, K18, and K56 of histone H3 [2,3,4,5]. Nonetheless, SIRT6 promotes different reactions on a wide range of substrates beyond histones [6]. In addition to protein deacetylation, SIRT6 catalyzes the protein deacylation of long-chain fatty acyl groups from the ε-amino groups of lysines and the mono-ADP-ribosylation of lysine and arginine residues of chromatin silencing DNA repair proteins [7].

SIRT6 expression is almost ubiquitous, with the highest levels detected in skeletal muscle, heart, brain, liver, kidney, and thymus [8,9]. SIRT6-catalyzed deacetylation is associated with compaction of chromatin and consequent transcriptional repression, as well as response to DNA damage. Notably, recent reports indicated that the SIRT6 deacetylase catalytic activity is 100 to 1000 times lower than that of the most active SIRTs [10]. The deacylase efficiency of SIRT6 has been shown to be higher compared to deacetylation, which can be in turn activated by endogenous ligands such as free fatty acids (FFA) [11,12]. Indeed, in vitro demyristoylation activity is roughly 300 times higher than deacetylation. On the other hand, most of SIRT6 cellular functions described to date are related to its deacetylation activity, rather than deacylation, which has been proven in the case of TNF-α [12] and R-Ras2 [13]. These features, along with the ability of SIRT6 to catalyze mono-ADP-ribosylation, depict a complicated picture of its biological functions and related phenotypes.

The capability of SIRT6 to regulate different molecular pathways is pivotal to maintain cellular homeostasis [6]. Upon DNA damage, an increase of SIRT6 levels determines an improvement of chromatin accessibility recruiting several DNA repair factors, such as 53BP1, BRCA1, and RPA to the breakpoint [14]. SIRT6 modulates double strand break (DSB) repair activating both non-homologous end-joining (NHEJ) and homologous recombination (HR), through the interaction with different proteins involved in these molecular pathways [15]. For instance, under oxidative stress SIRT6 associates with the poly[ADP-ribose]polymerase PARP1 and catalyzes its mono-ADP-ribosylation, thereby stimulating its activity and resulting in improved DSB repair [16].

SIRT6 is also involved in the base excision repair (BER) process in a PARP1-dependent manner [17] and contributes to genome and telomeres integrity in mammalian cells through the interaction with the DNA glycosylase MYH and the endonuclease APE1 [18]. This is in line with earlier studies indicating the requirement of SIRT6 for genome stability and telomeric maintenance in human cells [19]. Through H3K9 deacetylation at telomeres, SIRT6 facilitates the binding of WRN, the protein that is mutated in Werner syndrome, thus promoting proper telomeric function and metabolism [2,20]. Recent studies also indicated that SIRT6 interacts with telomere repeat binding factor 2 (TRF2), a critical protein for telomere maintenance and DNA damage response, catalyzing its deacetylation, leading to ubiquitination and, consequently, proteolysis [21].

SIRT6 is also able to bind at the 5′-UTR of long interspersed element 1 (L1) retrotransposons. Here, it mono-ADP-ribosylates the nuclear corepressor KAP1, thereby facilitating its interaction with the heterochromatin factor HP1α and ensuring genome stability through L1s packaging into transcriptionally inaccessible heterochromatin [22].

Given the requirement of NAD^+^ for their activity, SIRTs have been regarded as pivotal proteins connecting metabolism to cell physiology [23]. Loss-of-function studies performed in mouse models indicated the crucial roles that SIRT6 plays for organism well-being. Indeed, SIRT6-deficient mice displayed alteration of glycolysis and genomic instability, ultimately leading to premature aging and shortened lifespan [24,25,26].

SIRT6 also plays an important role in the regulation of inflammatory response, acting both as an activator and as a repressor. For instance, it promotes inflammation through TNF-α demyristoylation at K19 and K20, triggering its secretion by immune cells [12], while acylated TNF-α would be kept and finally degraded in lysosomes. In addition, SIRT6 action determines also upregulation of TNF-α and the pro-inflammatory cytokine IL-8 [27]. Conversely, it blocks the expression of pro-inflammatory factors such as NF-κB [28,29] and IL-6 [30], thereby suppressing the inflammatory mechanisms.

Given its multitasking biological roles in genome maintenance, metabolism, and inflammation, it is not surprising that SIRT6 has a critical role in the regulation of cell fate determination and apoptosis.

One of the main features of tumor cells is their ability to escape from programmed cell death (PCD), proceeding towards unregulated proliferation and consequent malignant transformation [31]. Due to the role played by SIRT6 in the regulation of cellular homeostasis, mounting evidence points towards its involvement in cancer.

SIRT6 has been found to modulate the expression of both pro- and anti-apoptotic factors, thus influencing cell survival and affecting cancer development in a context-dependent manner. In response to DNA damage, SIRT6 induces apoptosis via mono-ADP-ribosylation of p53 and p73 in several cancer cells, but not in normal or non-transformed cells [32]. Therefore, SIRT6 shows a dichotomous role in cancer, acting both as tumor promoter and suppressor in a cell context-dependent fashion [33,34,35]. SIRT6 role in cancer is reminiscent of other sirtuins such as SIRT2, which has been shown to act as either tumor suppressor or promoter in breast cancer, depending on tumor grade [36]. Likewise, SIRT2 may influence sensitivity to chemotherapeutics positively [37] or negatively [38], depending on the cancer type.

Many details connecting the biochemical activity of SIRT6 and the observed phenotypes in both physiological and pathological states are still missing, thus motivating further investigations. Chemical inactivation of SIRT6 through small molecules represents an important way to assess its biological functions. Moreover, the recent discoveries indicating that SIRT6 may also be activated by endogenous ligands encouraged the development of SIRT6 activators. Both activators and inhibitors represent valuable tools to study the functions of this multifaceted enzyme. In addition, they can act as lead compounds for the development of therapeutics for the treatment of diseases such as cancer, diabetes, obesity, and neurodegeneration. Cancer encompasses a vast number of different diseases characterized by a diverse and complex subset of biochemical features. Therefore, depending on the specific type of cancer, SIRT6 activation or inhibition may be beneficial.

In this review, we scrutinize the functions of SIRT6 in the regulation of cell death and cancer. We also focus on the most relevant SIRT6 activators and inhibitors, which may be used as tools to elucidate on SIRT6 physiological and pathological roles and may also represent potential therapeutics for SIRT6-related diseases.

## 2. Regulation of SIRT6 Expression and Activity

Several factors regulate SIRT6 expression and activity at transcriptional and post-transcriptional level, influencing its role on tumor initiation and progression. The transcription factor AP-1 induces transcription of SIRT6 through its c-Fos subunit, which directly binds to SIRT6 promoter. This correlation has been discovered in hepatocellular carcinoma (HCC), whereby c-Fos-mediated SIRT6 transcriptional activation initiates a tumor-suppressor pathway that will be explained in detail in the following section [39]. In contrast, the binding of the transcription factor E2F1 to SIRT6 promoter region blocks SIRT6 transcription under both normoxia and hypoxia conditions [40]. Similarly, PARP1 seems to downregulate SIRT6 expression since treatment with its inhibitor PJ-34 results in augmented levels of SIRT6 mRNA [41].

The expression and activity of SIRT6 is also modulated by the microRNA system. In particular, miR-33a, miR-33b and miR-34a were shown to decrease mRNA and protein levels of SIRT6 in different cell types [42,43,44,45,46]. In addition, SIRT6 and miR-122 negatively regulate their expression in a reciprocal way. miR-122, the most abundant hepatic miRNA, binds to the 3′-UTR of SIRT6 hence reducing its levels, while SIRT6 downregulates miR-122 through H3K56 deacetylation at its promoter [47]. Notably, SIRT6 and miR-122 oppositely modulate the transcription of the same genes involved with metabolism and fatty acid oxidation [47]. Similarly, SIRT6 and miR-125b negatively regulate each other, and miR-125b was shown to interact with 3′-UTR of SIRT6, directly suppressing its expression [48]. Finally, miR-766 and SIRT6 were shown to negatively regulate each other in a feedback manner and this mechanism is relevant in the context of aging cells reprogramming [49].

SIRT6 functions are also regulated at a post-translational level, through modifications and key interactions with other proteins (Table 1). For instance, AKT1-mediated phosphorylation of SIRT6 at Ser338 triggers its ubiquitination by MDM2, finally leading to proteasomal degradation [50]. Notably, cyclic AMP (cAMP) decreases SIRT6 levels through activation of PKA which in turn mediates the inhibition of the Raf-MEK-ERK pathways, finally leading to SIRT6 ubiquitination. In addition, PKA activates the transcription factor CREB, which in turn decreases SIRT6 expression [51]. On the other hand, the ubiquitin ligase CHIP catalyzes non-canonical SIRT6 ubiquitination at Lys170, thus preventing its canonical ubiquitination and protecting the protein from proteasomal degradation [52]. In addition, SIRT6 was demonstrated to be a substrate of the ubiquitin-specific peptidase USP10 which de-ubiquitinates SIRT6, thereby protecting it from proteasome-dependent degradation [53]. Finally, SIRT6 was found to interact with the SUMO-conjugating enzyme UBC9 which catalyzes its conjugation with SUMO1. SIRT6 SUMOylation positively regulates H3K56 deacetylation but, has no influence on H3K9. This modification promotes SIRT6 binding to c-Myc, and consequent occupancy at c-Myc target genes loci. SIRT6 then deacetylates H3K56Ac in these regions, thereby leading to silencing of c-Myc target genes [54].

## 3. Role of SIRT6 in Cancer

Numerous studies aimed at analyzing the pathways that are dysregulated in cancer have been focusing on the characterization of different molecular targets acting on DNA repair and cell death. Through their modulation, it is possible to control three important processes in tumor development such as initiation, progression and metastatization. SIRT6 prevents genome instability, maintains telomere integrity, facilitates DNA repair, and regulates metabolic homeostasis, thereby may have a pivotal role in carcinogenesis [6].

**Figure 1:**
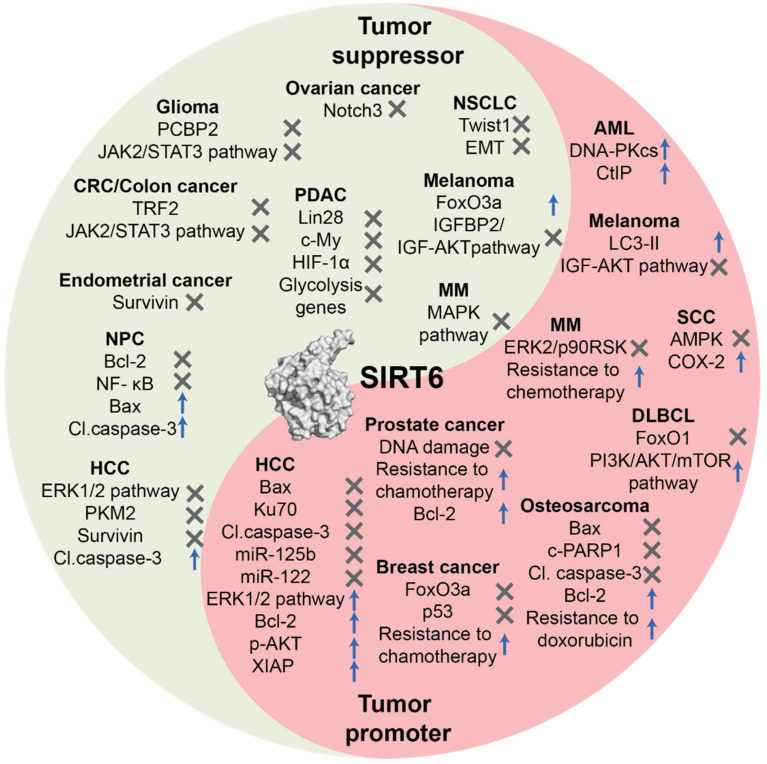
The contrasting functions of SIRT6 in tumorigenesis. SIRT6 acts both as tumor suppressor and tumor promoter in different contexts, depending on tissue types and the stage of cancer. Surface representation of SIRT6 crystal structure was retrieved from the Protein Data Bank (PDB ID: 3ZG6) and rendered with PyMol (Schrödinger).

An increasing number of studies reported an altered expression of SIRT6 in cancer, both at gene and protein levels. SIRT6 in tumorigenesis can be considered a double-edged sword, acting either as tumor promoter or suppressor depending on the biological context (Figure 1), and several reports underlined the connection between its action and the modulation of cell death in cancer [32,55].

### 3.1. Tumor Suppression Function

The analysis of different databases, such as the Cancer Cell Line Encyclopedia [56], reveals that SIRT6 expression is suppressed in several types of cancer, suggesting a tumor suppressor role for this enzyme. In addition, many SIRT6 point mutations able to alter and often impair its biological and enzymatic functions were discovered in tumors, ultimately leading to metabolic changes and transformation [33].

In ovarian cancer tissues, SIRT6 expression levels are lower than in the non-transformed counterparts [57]. Moreover, SIRT6 overexpression in ovarian cancer cells inhibits proliferation and the expression of Notch3, a prognostic factor for ovarian serous carcinoma [58]. A similar effect was observed also in glioma cells in which SIRT6 suppresses the expression of poly(C)-binding protein 2 (PCBP2) through H3K9 deacetylation at its promoter, hence blocking tumor cell growth [59,60]. Further evidence suggests the tumor suppressor role of SIRT6 in other types of cancer such as hepatocellular carcinoma (HCC) [61], lung cancer [62] and nasopharyngeal carcinoma (NPC) [63], where SIRT6 has been found downregulated at gene level compared to normal tissues. In HepG2 cells, SIRT6 overexpression impairs cancer proliferation through the inhibition of ERK1/2 signaling and promotes apoptosis by inducing increased levels of cleaved caspase-3 [61].

A negative correlation between SIRT6 and the nuclear glycolytic enzyme pyruvate kinase M2 (PKM2) was discovered in a HCC. PKM2 has non-metabolic oncogenic functions and is directly involved in metastatization. In hepatocellular carcinoma tissues reduced levels of SIRT6 were observed, along with high levels of acetylated PKM2 at residue K433. These findings highlighted a molecular mechanism by which SIRT6 deacetylates PKM2 at K433, triggering its nuclear export and blocking its oncogenic functions [64].

In non-small cell lung cancer (NSCLC), the inhibition of proliferation mediated by SIRT6 is the result of the suppression of Twist1 expression, a key player involved in two different tumor processes such as metastatization and epithelial-mesenchymal transition (EMT) [62]. Lack of SIRT6 in pancreatic ductal adenocarcinoma (PDAC) determines hyperacetylation of H3K9 and H3K56 at the promoter of the oncogene Lin28b, along with c-Myc recruitment, resulting in the enhancement of cancer progression and metastatization [65]. PDAC is also characterized by increased expression of glycolytic genes which is correlated with SIRT6 downregulation [26]. Indeed, SIRT6 deacetylates H3K9 at glycolytic genes promoters [66] and co-represses the hypoxia-inducible factor 1α (HIF-1α). This protein facilitates the expression of glycolytic genes such as lactate dehydrogenase (LDH), pyruvate dehydrogenase kinase-1 (PDK1), phosphofructokinase-1 (PFK1), and the glucose transporter-1 (GLUT1) [66]. Through this action, SIRT6 exerts a tumor suppressor role as it blocks the so-called Warburg effect. This is an alteration in glucose metabolism common in cancer cells in which ATP is produced mainly through glycolysis, even in the presence of oxygen. This leads to quick production of energy to support fast cancer cell growth [67].

Colorectal cancer (CRC) is also characterized by SIRT6 downregulation and increased expression of glycolysis-related genes [26]. In addition, SIRT6 and TRF2 expression levels were inversely correlated in a cohort of CRC patients, suggesting a regulatory mechanism whereby SIRT6 induces degradation of TRF2, which is overexpressed during oncogenesis. Nonetheless, the consequences of SIRT6/TRF2 in the damage repair pathway and apoptosis remains to be further clarified [21]. As previously mentioned, the peptidase USP10 de-ubiquitinates SIRT6, thereby protecting it from proteasomal degradation. In line with this, USP10 expression correlates positively with SIRT6 expression and both proteins are downregulated in colon cancer. Additionally, USP10 blocks tumor formation via p53 and SIRT6-mediated degradation of c-Myc, thereby stopping cell cycle progression and cancer cell growth [53]. It is well known that JAK2/STAT3 signaling pathway is constitutively activated in most primary malignant cancers and its activation rate is positively related with tumor grade. A recent study showed that high levels of SIRT6 expression in colon cancer are associated with a better prognosis. Indeed, following the action of the non-coding RNA miRNA-34c-5p, JAK2/STAT3 pathway is activated, thereby negatively regulating SIRT6, inhibiting apoptosis, and inducing colon cancer growth [68]. As mentioned above, SIRT6 protein expression is reduced in glioma cell lines, where it negatively correlates with the expression of miR-33a. In this cancer model, SIRT6 restoration led to apoptosis through upregulation of Bax and cleaved caspase-8, along with downregulation of Bcl-2 and inhibition of the JAK2/STAT3 pathway. These events resulted in the reduction of glioma cancer cell survival [44].

In NPC, the NF-κB pathway is particularly active and is involved in the activation of anti-apoptotic proteins such as FLIP, c-IAP1/2 and XIAP, favoring cancer resistance and progression [69]. Notably, SIRT6 was found to be downregulated in NPC and its restoration led to decreased levels of NF-κB and anti-apoptotic factor Bcl-2, along with augmented expression of pro-apoptosis mediators Bax (Bcl-2 associated X protein) and cleaved caspase-3 [63].

Mounting evidence points towards a functional correlation between the activities of SIRT6 and the anti-apoptosis factors. In several cancer types, low levels of SIRT6 were associated with marked expression of the pro-survival protein survivin, a condition that correlates with tumor aggression and poor patient survival [39,70]. In a liver cancer mouse model SIRT6 has a tumor suppression effect, repressing the transcription of survivin at two levels: through H3K9 deacetylation at its promoter and through NF-κB deacetylation, which impairs its binding to survivin promoter [39]. The same molecular mechanism has also been described in endometrial cancer cell lines [70], thus highlighting the pro-apoptotic role of SIRT6 via survivin inhibition.

In melanoma, SIRT6 has been shown to act as both a tumor suppressor and promoter. A recent investigation indicated that SIRT6 expression is positively correlated with FoxO3a expression [71]. FoxO3a is a tumor suppressor [72] involved in the positive regulation of apoptosis [73,74,75], in the protection against oxidative stress [76], and also in the cholesterol biosynthesis regulation along with SIRT6 [77,78]. In addition, FoxO3a negatively regulates the expression aerobic glycolytic genes. SIRT6 overexpression was shown to augment FoxO3a levels, reducing the levels of glycolytic genes and cancer cell proliferation [71]. SIRT6 activity also influences the IGF-AKT pathway. Strub et al. showed that SIRT6 downregulation increases H3K56 acetylation at the promoter of Insulin-like Growth Factor Binding Protein 2 (IGFBP2), thereby increasing its expression levels. IGFBP2 then activates the Insulin Growth Factor 1 receptor (IGF-1R) and downstream signaling of the anti-apoptotic protein AKT (or protein kinase B, PKB), thus promoting melanoma cell survival and drug resistance to MAPK signaling inhibitors [79].

### 3.2. Tumor Promoter Function

An increasing number of studies report that SIRT6 expression is significantly associated with both solid and hematological human cancer types such as head and neck squamous cell carcinoma [80], HCC [55,81,82,83], prostate cancer [84], breast cancer [85], skin squamous cell carcinoma (SCC) [45,86], melanoma [87,88,89,90], diffuse large B-cell lymphoma (DLBCL) [91], and acute myeloid leukemia (AML) [92] highlighting its role in tumorigenesis as tumor promoter.

SIRT6 oncogenic role was extensively studied in HCC, where it was found to be upregulated in a subset of HCC tissue and cell lines. Its high expression levels were associated with increased tumor grade and metastatization [81,82]. Indeed, it facilitates the EMT, which is one of the main processes involved in metastasis. This outcome is obtained through deacetylation of Beclin-1, triggering autophagic degradation of E-cadherin, a pivotal cell adhesion protein [81]. In another study performed on HCC tissues, SIRT6 was shown to deacetylate H3K9 at the promoter of the apoptotic activator Bax, resulting in evasion from apoptosis [82]. Conversely, SIRT6 depletion determined hyperacetylation of Bax promoter, with the recruitment of several transcription factors (e.g., p53) that in turn activate Bax and its downstream effectors [93], resulting in an activation of death pathway. Moreover, mRNA levels of SIRT6 and Bax were negatively correlated in human samples. These results were corroborated by experiments performed in mouse xenografts, where SIRT6 knockout impaired tumor growth and led to apoptosis [82]. It has also been reported that the inhibition of the molecular pathway of Bax is the result of the association between SIRT6 and Ku70, a subunit of the complex involved in DNA repair [94]. Although SIRT6 is a nuclear protein, during cell cycle progression it can translocate to the cytosol and catalyze Ku70 deacetylation, consequently blocking Bax-mediated intrinsic apoptotic pathway [94]. Novel insights about the oncogenic role of SIRT6 in HCC indicated that the SIRT6-mediated activation of the ERK1/2 pathway can promote cancer cell proliferation and invasion. In addition, SIRT6 overexpression induces upregulation of Bcl-2, while downregulates Bax and cleaved caspase-3, consequently inhibiting the intrinsic apoptotic pathway [55]. Moreover, SIRT6 deacetylates the anti-apoptotic factor AKT, which in turn promotes its phosphorylation and increases its activity. AKT then phosphorylates X-linked inhibitor of apoptosis protein (XIAP) thereby inducing evasion from apoptosis and cancer cell proliferation [83]. Furthermore, the oncogenic role of SIRT6 in HCC was also elucidated by studying the relations with miRNA levels. Indeed, a high expression of SIRT6 is associated with low levels of miR-125b with consequent inhibition of the apoptotic pathway [48]. The previously mentioned reciprocal regulation between SIRT6 and miR-122 has been described as a crucial mechanism to control hepatic metabolic functions. Intriguingly, the loss of this reciprocal negative regulation in HCC patients, followed by high expression of both players, correlates with hepatic good prognosis, suggesting that these two factors may serve as biomarkers for HCC prognosis [47].

The tumor promoting role of SIRT6 has also been investigated in other cancer types, where reduction of SIRT6 levels is associated to a better response to chemotherapeutics [84,85,95]. In metastatic prostate cancer cell lines, SIRT6 knockdown improves chemotherapy sensitivity, with increased DNA damage and consequent cell cycle arrest in G1 along with Bcl-2 downregulation and apoptosis induction [84]. In breast cancer cells, high levels of SIRT6 are associated with resistance to epirubicin and paclitaxel. Indeed, SIRT6 activates DNA repair pathways in response to epirubicin-induced damage. SIRT6 also antagonizes the activity of the tumor suppressors p53 and FoxO3a both directly via deacetylation, and indirectly through suppression of their expression [85]. In osteosarcoma cell lines, SIRT6 knock-down has been shown to potentiate the effect of conventional chemotherapeutics inducing block of cancer cell proliferation and cell death [95]. SIRT6 inhibition, together with the action of doxorubicin, results in an increase of apoptotic response with a higher expression of Bax and cleaved forms of PARP1 and caspase-3, along with the downregulation of Bcl2 [95].

The tumor promoting capacity of SIRT6 has also been demonstrated in skin SCC, where SIRT6 is upregulated [86]. Following exposure to UV-B radiation, the activation of AKT pathway induces an increase of SIRT6 levels which promotes COX-2 expression through repression of AMP-activated protein kinase (AMPK) signaling, finally leading to cancer cell survival and proliferation [86]. In addition, SIRT6 is silenced by miR-34a, a miRNA critical for squamous cell differentiation which is suppressed in skin and oral SCCs. Notably, SIRT6 downregulation is sufficient to reactivate the pro-differentiation effects of miR-34a and to reduce the cell proliferation potential [45].

As mentioned above, SIRT6 has also tumor promoting functions in melanoma. Indeed, it was shown to be upregulated in both cell lines and clinical tissue samples of human melanoma, and its knockdown resulted in cell cycle arrest at G1, senescence and impairment of cell growth [87]. In addition, SIRT6 was found to positively modulate autophagy as its knockdown was associated with decreased conversion of the microtubule-associated protein 1A/1B-light chain 3 (LC3) from LC3-I to the phosphatidylethanolamine-conjugated form LC3-II, a crucial autophagosome initiator [87]. In agreement with this, a CRISPR/Cas9-mediated knockout of SIRT6 determined antiproliferative effects both in vitro and in vivo [88]. Notably, another study indicated that SIRT6 has a dichotomous effect in melanoma, suppressing the growth of primary melanoma while promoting the development of metastatic melanoma both in vitro and in vivo. This action was shown to be correlated with the expression of autophagy biomarkers, suggesting an autophagy-dependent mechanism played by SIRT6. As previously mentioned, SIRT6 inhibits the IGF-AKT pathway, which is also responsible for autophagy suppression [89]. Mechanistically, autophagy aids the degradation of toxic protein at initial stages, but also reduces cancer cell susceptibility to stress and promotes the development of established tumors, hence explaining SIRT6 dual role in melanoma [90].

SIRT6 was found to be overexpressed also in DLBCL where its levels are related to poor prognosis. In this setting, SIRT6 activates the PI3K/AKT/mTOR pathway, thus facilitating cancer progression [91]. Moreover, SIRT6 inhibition or knockdown result in reduced drug resistance and apoptotic cell death induction [91]. Indeed, in LY1 and LY8 follicular lymphoma cells, SIRT6 knockdown promotes the apoptotic cascade with high levels of cleaved PARP and reduction of several phosphorylated targets of PI3K pathway such as PI3K(p110), Akt (Ser473), and p-mTOR, and a significant increase of the FoxO1 oncosuppressor levels [91].

SIRT6 is also upregulated in AML if compared with normal CD34+ hematopoietic progenitors [92]. Following genotoxic stress, SIRT6 is recruited to DNA damage sites where it activates through deacetylation DNA-PKcs and CtIP, constitutively acetylated in AML cells, consequently promoting DNA repair which in turn supports cancer cell survival. Conversely, the overexpression of SIRT6 catalytic mutant (H133Y) in AML determines more pronounced anticancer effects [92]. SIRT6 is also highly expressed in MM cells, in which it is associated with adverse prognosis [96]. However, in this case, the authors propose that SIRT6 higher expression is an adaptive response to genomic instability and describe a situation where SIRT6 exerts a dual role. Indeed, SIRT6 interacts with the transcriptional activator ELK1 and deacetylates H3K9 at the promoters of ERK signaling-related genes, thus downregulating the expression of mitogen-activated protein kinase (MAPK) pathway genes and inhibiting proliferation consistent with a role as tumor suppressor, rather than promoter. In line with this, its depletion in MM xenografts enhances cancer growth. On the other hand, SIRT6 knockdown increased ERK2 expression and p90RSK phosphorylation thus facilitating DNA repair and, consequently, resistance to DNA damaging agents. In line with this, low SIRT6 levels sensitise MM xenografts to DNA-damaging agents such as doxorubicin [96]. This study provides important insights into the crosstalk between SIRT6 activity and cancer cell proliferation. Indeed, whilst showing a tumor-suppressor role in this type of cancer, the authors suggest that SIRT6 inhibition may enhance sensitivity to chemotherapeutics consequently improving patient prognosis. 

A summary of SIRT6 role in cancer regulation is provided in Table 2.

## 4. Pharmacological Modulation of SIRT6

The discovery that SIRT6 deacetylase activity is enhanced by FFA, along with its positive role in aging and cell metabolism stimulated research groups towards the development of SIRT6 activators. Conversely, the dual role of SIRT6 in cancer, cell survival and inflammation has also motivated the development of SIRT6 inhibitors.

The possibility of either activating or inhibiting SIRT6 in a context-dependent manner paves the way for personalized pharmacology. From a wider perspective, highly potent and selective SIRT6 modulators (both activators and inhibitors) allow to better scrutinize the molecular details of its activity, and further validate this complex enzyme as a potential pharmacological target.

In the following sections, we discuss the most relevant SIRT6 activators and inhibitors that have been indicated to impact cancer progression and cell death so far.

### 4.1. SIRT6 Activators

**Figure 2:**
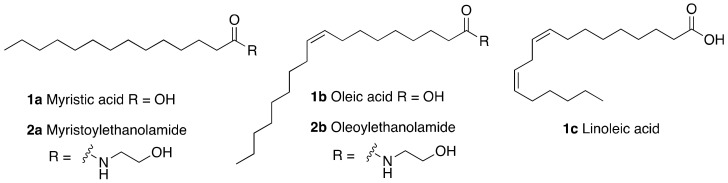
Free fatty acid SIRT6 activators and derivatives.

To give a benchmark for the evaluation of SIRT6 activators (Table 3), it is worth mentioning the initial reports describing FFA-mediated SIRT6 activation. Myristic acid (**1a**), a 14-carbons fatty acid, increased SIRT6 deacetylase activity up to 10.8 times, with an EC_50_ (concentration able to induce 50% of maximal activation) of 246 μM and a 35-fold maximum increase in catalytic efficiency (*k_cat_/K_m_*), indicating enhanced affinity of SIRT6 for its substrate [11]. The unsaturated 18-carbons FFAs oleic (**1b**) and linoleic acid (**1c**) (Figure 2) displayed EC_50_ values of 90 μM and 100 μM, yielding up to 5.8 and 6.8 times SIRT6 maximum activation, respectively [11]. Intriguingly, the ethanolamine derivatives of myristic acid and oleic acid, myristoylethanolamide (MEA, **2a**) and oleoylethanolamide (OEA, **2b**) showed up 2-fold SIRT6 activation and EC_50_ values of 7.5 μM and 3.1 μM, respectively [97].

**Figure 3:**
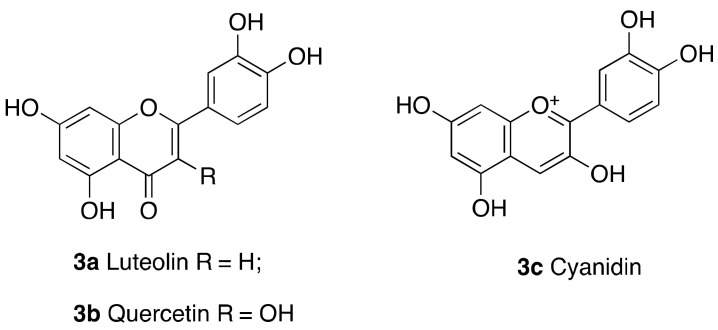
Flavonoid-based SIRT6 modulators.

Early studies on flavonoids indicated that the natural products luteolin (**3a**) and quercetin (**3b**) (Figure 3) presented a dual effect on SIRT6 activity. At low concentrations, they acted as inhibitors (IC_50_(**3a**) = 1.9 μM; IC_50_(**3b**) = 24 μM). Conversely, at high concentrations they enhanced SIRT6 deacetylase activity, with a 6-fold maximum activation and EC_50_ value of 270 μM in the case of luteolin, and a 10-fold maximum activation and EC_50_ value of 990 μM in the case of quercetin [97]. It should be noticed that the polyphenol structure of these molecules makes them amenable for multi-target interactions and promiscuous activities. Interestingly, the anthocyanidin cyanidin (**3c**), which showed 55-fold activation of SIRT6 activity and EC_50_ = 460 μM, increased also SIRT6 expression levels in a dose-dependent fashion and exhibited important effects in colon adenocarcinoma Caco-2 cells. Indeed, it modulated the expression of SIRT6-associated genes with a dose-dependent increase of FoxO3a expression and decrease of Twist1 and GLUT1 levels [98].

Crystal structures of SIRT6 in complex with quercetin and cyanidin (PDB IDs: 6QCD and 6QCH, respectively) indicate that these molecules share the same binding at the distal end of acyl binding hydrophobic pocket, with weak interactions with the loop that lids the channel and key hydrogen bonds with Pro62 and conserved water molecules [105].

**Figure 4:**
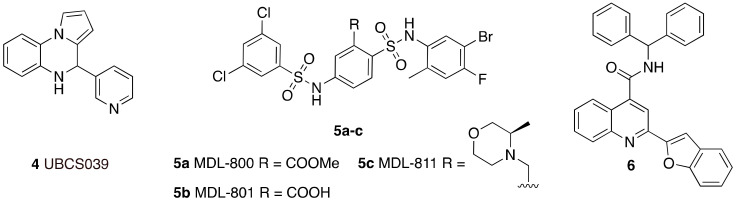
Synthetic SIRT6 activators.

The first synthetic SIRT6 activator reported in literature was the compound UBCS039 (**4**) (Figure 4), which exhibited an EC_50_ of 38 μM and 3.5-fold maximum activation of SIRT6 deacetylase activity [99]. Notably, UBCS039 specifically binds to SIRT6, has no significant influence on SIRT1-2-3 deacetylation activities, while stimulates up to 2-fold the SIRT5-mediated desuccinylation, which is the physiologically dominant activity of this enzyme. The UBCS039-SIRT6 co-crystal (PDB ID: 5MF6) indicates that UBCS039 shares a similar binding site with quercetin and cyanidin at the edge of the acyl channel pocket. Further comparison with the SIRT6-Myr-H3K9 peptide co-crystal reveals that UBCS039 overlaps with the last seven carbons of the acyl chain, in line with experimental evidence suggesting competition for the same binding site, although the affinity of the myristoylated peptide is much higher. Evaluation on histones extracted from calf thymus and HeLa nucleosomes also indicated the stimulation of H3K18 deacetylation by UBCS039 [99]. Cell-based studies revealed that UBCS039 activates SIRT6 and decreased H3K9 and H3K56 acetylation levels in various cancer cell lines such as NSCLC, fibrosarcoma, colon and epithelial cervix carcinoma [100]. In NSCLC and epithelial cervix carcinoma cell lines, UBCS039-mediated SIRT6 activation led to time-dependent autophagosome accumulation and autophagy activation. Cell growth inhibition and apoptosis induction was also observed, indicating an autophagy-associated cell death mechanism. In addition, UBCS039 promoted the accumulation of reactive oxygen species (ROS), in line with a recent study suggesting an important role of SIRT6 in oxygen consumption and consequent ATP production [106]. This study indicates that excessive autophagy stimulation is lethal for cancer cells and paves the way for therapies based on pharmacological activation of SIRT6 to exploit this mechanism [100].

Another synthetic molecule showing efficacy towards SIRT6-mediated pathways in cells is MDL-800 (**5a**) (Figure 4) [101]. With its EC_50_ value of 10.3 μM and 22-fold maximum SIRT6 deacetylase activity activation, MDL-800 is one of the most potent activators discovered so far. MDL-800 displayed selective activation towards SIRT6, when compared with 18 diverse HDAC family members and decreased H3K9 and H3K56 acetylation in HeLa-extracted nucleosomes. MDL-801 (**5b**) is the carboxylic acid derivative of MDL-800, it has a slightly better SIRT6 activating potency (EC_50_ = 5.7 μM) but is not cell permeable. Zhang and colleagues solved the co-crystal structure of SIRT6 bound to ADP-ribose, H3K9 myristoylated peptide, and **5b**. Given the structural similarities between MDL-801 and MDL-800, the observed features are likely shared between the two compounds. MDL-801 interacts in a different pocket compared to UBCS039 as it fits in a surface-exposed region of the hydrophobic channel, distal to UBCS039 binding site [101]. At cellular level, MDL-800 caused decreased acetylation of H3K9 and H3K56 in three different HCC cell lines. This phenotype is the result of SIRT6 activation and finally led to inhibition of proliferation and cell cycle arrest, with IC_50_ values for cell growth (IC_50-growth_) between 18.6 μM and 24 μM, depending on the specific cell line. These results were corroborated by experiments in mouse xenograft models, where MDL-800 suppressed HCC tumor growth through SIRT6 activation. A recent investigation expanded these results and showed that MDL-800 inhibits the proliferation of 12 NSCLC cell lines. Cell cycle arrest at the G_0_/G_1_ phase was observed in NSCLC HCC827 and PC9 cells, consistent with studies indicating the role of SIRT6 in cell cycle regulation [3,53]. Notably, it exhibited synergistic activity with epidermal growth factor receptor tyrosine kinase inhibitors (EGFR-TKIs) in osimertinib-resistant HCC827 and PC9 cells and in patient-derived primary tumor cells. Moreover, MDL-800 suppressed tumor growth in HCC827 cell-derived xenograft nude mice and caused H3 deacetylation and downregulation of p-MEK and p-ERK in tumor tissues [102]. When tested in old murine-derived induced pluripotent stem cells (iPSCs), MDL-800 improved genome integrity through the activation of both NHEJ and BER, in line with the SIRT6 pivotal role in controlling DNA repair pathways [107]. In addition, it improved the differentiation potential of iPSCs, consistently with the SIRT6 role in the modulation of both iPSCs and ESCs [108,109].

Optimization of MDL-800 led to compound MDL-811 (**5c**) with improved activity (EC_50_ = 5.7 μM) and bioavailability in C57BL/6J mice (F%_MDL-800_ = 71.33% vs. F%_MDL-811_ = 92.96%) [103]. Like its lead compound, MDL-811 is specific towards SIRT6 deacetylase activity and reduced the acetylation levels of H3K9, H3K18, and H3K56 in nucleosomes extracted from HeLa cells and in HEK293T cells. When evaluated in CRC cell lines, a type of tumor characterized by heavy downregulation of SIRT6, MDL-811 caused a dose-dependent decrease of H3K9Ac, H3K18Ac, and H3K56Ac levels and antiproliferative effects associated with marked G0/G1 cell cycle arrest. MDL-811 also suppressed CRC growth in patient-derived organoids and showed anti-tumor efficacy in cell line-derived and patient-derived xenograft models, as well as in a spontaneous CRC mouse model [103]. Mechanistically, the cytochrome P450 family member CYP24A1, that is aberrantly overexpressed in CRC [110,111], was identified as a new target gene of SIRT6. MDL-811 suppressed CRC proliferation synergistically with vitamin D_3_, which is both a substrate and transcriptional regulator of CYP24A1 and had previously shown anti-tumor efficacy in CRC [112,113]. These features depict MDL-811 as a potential good candidate for clinical studies.

A virtual screening campaign led to the discovery of the compound **6** (Figure 4) as a potent and selective small molecule activator of SIRT6 [104]. This molecule was optimized starting from an initial hit identified using the SIRT6-UBCS039 complex (PDB ID: 5MF6) as model [99]. Compound **6** enhanced both SIRT6 deacetylase and deacylase activities, with EC_50_ values of 5.35 μM and 8.91 μM for deacetylation and demyristoylation, respectively. The isoform selectivity was tested over HDAC1-11 and SIRT1-3 showing no activity towards any of these enzymes. According to docking experiments compound **6** binds SIRT6 more towards the distal end of the hydrophobic channel compared to UBCS039, which may justify its augmentation of SIRT6 deacylase activity. Compound **6** suppressed the proliferation of pancreatic ductal adenocarcinoma (PDAC) cells and caused cell cycle arrest in G2. These results were confirmed in vivo as **6** exhibited anti-tumor activity in a human pancreatic tumor xenograft mouse model associated with decrease of H3K9 acetylation levels. In addition, a preliminary study in male Sprague-Dawley rats indicated a promising pharmacokinetic profile, although the bioavailability was only 4%. Notwithstanding the low bioavailability, **6** has a good pharmacokinetic profile and is the most potent SIRT6 activator described so far. With its low micromolar EC_50_ and in vivo efficacy, it is an ideal lead compound for further development of potent and selective activators of SIRT6 with improved bioavailability that might be promoted to the clinical phase.

### 4.2. SIRT6 Inhibitors

Given the double-faced involvement of SIRT6 in cancer and inflammation, the inhibition of SIRT6 in specific contexts may also represent a successful strategy for cancer treatment. Indeed, inhibitors may target different SIRT6-mediated pathways contributing to cancer progression such as DNA repair mechanisms, cell differentiation and inflammatory response (Table 4).

**Figure 5:**
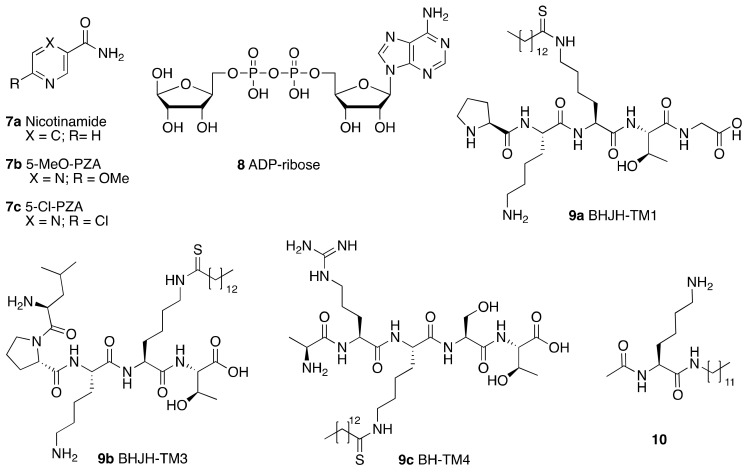
Product- (**7**–**8**) and substrate-based (**9**–**10**) SIRT6 inhibitors.

Product-based inhibitors such as nicotinamide (**7a**) and its derivatives, as well as ADP-ribose (**8**) (Figure 5) presented IC_50_ values in the mid-micromolar range, although the selectivity was absent or not tested. Nicotinamide showed IC_50_ values for the demyristoylation activity between 73 μM and 184 μM depending on the assay conditions [120,121]. Nicotinamide derivatives based on pyrazinamide showed improved SIRT6 inhibitory activity: 5-MeO-PZA (**7b**) and 5-Cl-PZA (**7c**) had IC_50_ values of 40.4 μM and 33.2 μM, respectively [122]. ADP-ribose (**8**) also inhibits SIRT6 activity and shows higher potency than nicotinamide with IC_50_ values of 74 μM (deoctanoylation) and 89 μM (demyristoylation), compared to values of 150 μM and 120 μM, respectively, for nicotinamide [123].

Another class of inhibitors directly related to the SIRT6 enzymatic mechanism of action are N^ε^-thioacyl-lysine-containing peptides, which lock the catalytic cycle at the first step, i.e., the nucleophilic attack to the (thio)carbonyl of the acyl group [124]. Thiomyristoyl peptides BHJH-TM1 (**9a**), BHJH-TM3 (**9b**), and BH-TM4 (**9c**) (Figure 5) are based on known SIRT6 substrates (i.e., TNF-α-K20, TNF-α-K19 and H3K9 peptides) [114]. Their IC_50_ values for demyristoylation were 2.8 μM, 8.1 μM and 1.7 μM, respectively, though they lacked selectivity due to the concomitant inhibition of SIRT1-3. **9c** was also tested in a deacetylation activity assay, showing an IC_50_ of 8.2 μM. Notwithstanding their peptide nature, these compounds were also active in HEK293T cells, displaying SIRT6 inhibition and increased TNF-α fatty acylation, with **9b** being the most potent. This result may be explained by the presence of a hydrophobic myristoyl group which can increase their cell permeability.

Another substrate-based compound targeting SIRT6 deacylase activities has been developed using a lysine residue as starting scaffold (**10**) (Figure 5) [125]. This molecule consists of a lysine where the amidic nitrogen is bound to a 12-carbon alkyl chain and the Cα amine is acetylated. Compound **10** inhibited SIRT6 deacetylation (IC_50_ = 95 μM) without isoform specificity as it also decreased SIRT1,2 activities with comparable potency. Interestingly, it enhanced demyristoylation (EC_50_ = 70 μM) and depalmitoylation (80% activation of at 100 μM), while still acting as an inhibitor for SIRT1 and SIRT2 deacylation. Nonetheless, MCF-7 breast cancer cell lines treatment with **10** resulted in increased H3K9Ac levels, intensification of the activities of glycolysis enzymes and decreased TNF-α secretion. This phenotype is in line with SIRT6 involvement in downregulation of glycolytic enzymes and its ability in triggering TNF-α secretion [12,126]. The results of this study are rather surprising in light of the previously cited evidence that FFA increase the deacetylation activity SIRT6 and inhibit deacylation [11]. Based on in silico data, the authors speculate that the acetyl moiety bound to the Cα amine group may mimic the acetylated substrate, being close to NAD^+^, in accordance with the experimental evidence suggesting competition of **10** with the acetylated substrate, rather than with NAD^+^. Nevertheless, further experimental evidence is required to clarify the binding mode and to account for the differential SIRT6 modulation profile.

**Figure 6:**
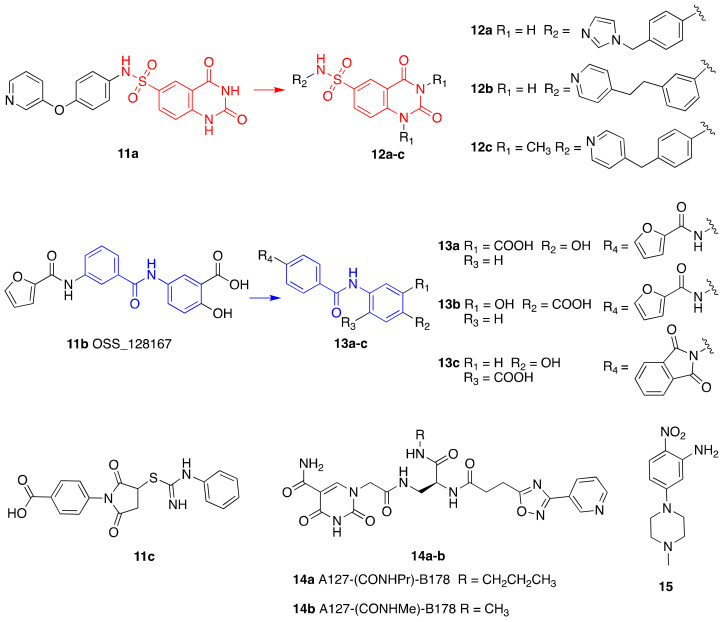
Synthetic SIRT6 inhibitors.

An in silico screening led to the identification of compounds **11a**–**c** (IC_50_s = 106 μM, 89 μM, and 181 μM, respectively) as the first SIRT6-inhibiting small molecules (Figure 6) [115]. Whilst **11a** showed mild selectivity over SIRT1, but not SIRT2, **11b** (later named OSS_128167) and **11c** were selective over both isoforms. The three compounds increased H3K9 acetylation in BxPC3 cells and induced GLUT1 upregulation and consequent increase of glucose uptake in L6 rat myoblasts and BxPC3 cells [66]. In addition, all compounds decreased TNF-α release. The effects of compounds **11a**–**c** are in line with the reported SIRT6 physiological roles and mimic the phenotypes observed following SIRT6 knockdown. Recently, **11a** showed improved glucose tolerance in a mouse model of type 2 diabetes mellitus, associated with reduced insulin, triglycerides, and cholesterol levels in plasma [127]. Compound **11b** was able to sensitize primary MM cells, as well as melphalan- and doxorubicin-resistant MM cell lines, to chemotherapeutics inducing DNA-damage. Additionally, the authors of this study showed that **11b** reduced recruitment of SIRT6 to DNA-damage sites [96]. When evaluated in DLBCL cells, that are characterized by overexpression of SIRT6, **11b** decreased cell viability and suppressed proliferation in a time- and dose-dependent manner. In line with this, induction of apoptosis along with cell cycle arrest at G2/M were observed. This compound showed also in vivo efficacy in a mouse xenograft model, where it decreased tumor growth and lowered the levels of the proliferative marker Ki-67 [91].

Compound **11a** was the lead molecule for the development of the series of quinazolinedione derivatives **12a**–**c** (Figure 6) [116], that are characterized by different substituents on the sulfonamide residue. These molecules showed improved SIRT6 inhibition (IC_50_(**12a**) = 60 μM; IC_50_(**12b**) = 37 μM; IC_50_(**12c**) = 49 μM), as well as isoform selectivity over SIRT1,2. Whilst all derivatives augmented H3K9 acetylation in BxPC3 cells, only compounds **12b** and **12c** determined higher glucose uptake in BxPC3 cells, as well as L6 rat myoblasts. Remarkably, compounds **12a** and **12b** sensitized BxPC3 cells to the chemotherapeutic agent gemcitabine and intensified the DNA damage and cell death induced by the PARP inhibitor olaparib in Capan-1 cells (a BRCA2-deficient pancreatic cancer cell line). These observations are consistent with previous findings suggesting that SIRT6 knockdown can improve the efficacy of chemotherapeutics [84].

Compounds **13a**–**c** (Figure 6) are the result of a ligand-based drug design approach that used **11b** as starting scaffold. They possess IC_50_ values of 34 μM, 22 μM, and 20 μM, respectively [117]. While **13c** was not cellularly active, **13a** and **13b** increased H3K9 acetylation and glucose uptake in human peripheral blood mononuclear cells (PBMCs). Compound **13b** displayed anti-proliferative effects in the same cell line. In addition, both **13a** and **13b** diminished TNF-α secretion and sensitized pancreatic cancer cells to gemcitabine [117].

A drug screening using DNA-encoded libraries designed for NAD^+^-binding pockets led to the identification of the SIRT6 inhibitors A127-(CONHPr)-B178 (**14a**) and A127-(CONHMe)-B178 (**14b**) (Figure 6), possessing IC_50_ values for demyristoylation in the low micromolar range (6.7 μM and 9.2 μM, respectively) [128]. Compound **14a** was selective over other sirtuins and was stable in serum after 72 h incubation. Like other inhibitors, **14a** caused dose-dependent decrease in the TNF-α levels. Moreover, treatment of primary human umbilical venous endothelial cells (HUVECs) with **14a** caused an increase of DNA-damage markers and telomere-dysfunction induced foci, similarly to what observed with SIRT6 knockdown [118].

Finally, the 1-phenylpiperazine derivative (**15**) (Figure 6) displayed selective and potent inhibition of SIRT6, with an IC_50_ of 4.93 μM in a peptide deacetylation assay [119]. When tested in BxPC-3 cells **15** augmented H3K9Ac and H3K18Ac levels, and enhanced GLUT-1 expression. Even more importantly, it had in vivo effects since it reduced the blood glucose content in a mouse model of type 2 diabetes, demonstrating promising drug-like properties.

## 5. Conclusions and Perspectives

Over the past decade, the elucidation of SIRT6 manifold functions has stimulated several studies on the connection between SIRT6 activity and both physiological and pathological conditions. Given the key role that SIRT6 plays in homeostasis regulation, it is not surprising that its function regulates cancer onset and progression. Its dual role in cancer is yet to be fully understood, however different reports point towards a context- and tissue-dependence. SIRT6-mediated DNA repair initially protects from tumor transformation, while promotes cancer cell survival at later stages. Importantly, the increase of SIRT6 levels in certain types of cancer may also represent an adaptation mechanism against genomic instability [96].

Several efforts have been dedicated to the improvement of anticancer therapies due to the emergence of resistance to chemotherapy, the low efficacy of different drugs and the high recurrence rate after surgery. Understanding the outcomes of different cellular pathways, such as the activation of programmed cell death and their involvement in tumorigenesis, will increase the efficacy of therapeutic strategies, strengthening the already well-known concept of personalized therapy. In the case of SIRT6, given its multifaceted role in cancer, the concept of personalized medicine becomes central: there is need for both activators and inhibitors, depending not only on the cancer type, but also likely on the stage of the disease. The modulation of cell proliferation and death, as well as the regulation of several factors linked to the tumor initiation, progression and metastatization, makes it a potential target for future personalized therapies.

The road to the discovery of potent and selective SIRT6 modulators is still at its infancy. Nonetheless, activators endowed with cellular activity such as UBCS039 (4), MDL-800 (5a), and MDL-811 (5c) have been described, with the two MDL compounds also showing in vivo efficacy. Notably, the UBCS039-SIRT6 co-crystal paved the way for structure-based discovery of compound 6, possessing anti-tumor activity both in vitro and in vivo. Amongst these activators, 5c and 6 represent the best lead compounds for the further optimization toward clinical candidates, most likely in the context of anti-cancer combination therapy.

As for the inhibitors, only one compound (11b) displayed anti-tumor activity in vivo. The structural optimization of 11b to increase its potency and PK properties represents a necessary step for the development of SIRT6 inhibitors with a solid therapeutic potential in cancer. Compound 15 had in vivo efficacy, although it was not tested in cancer models. Notably, this molecule is relatively simple and may act as lead compound for further optimization studies.

The elucidation of SIRT6-drug interactions at structural level will be crucial in the next future for the design of potent modulators. Novel molecules will help to dissect the details underpinning SIRT6 involvement in cancer but may also be proposed soon as drugs to be used in monotherapy or combined therapy to tackle different types of tumors.

## Figures and Tables

**Table 1:** Main SIRT6 interacting partners, modifications and influence on protein activity.

Interactor	Modification	Influence on SIRT6	Reference
AKT1	Ser338 Phosphorylation	Degradation	[50]
CHIP	Lys170 Ubiquitination	Stabilization	[52]
USP10	Deubiquitination	Stabilization	[53]
UBC9	SUMOylation	Increased H3K56 deacetylation	[54]

**Table 2:** Expression of SIRT6 in cancer and related biological functions.

Cancer Type	SIRT6 Expression	Cell Death Pathways and Regulators	Reference(s)
Ovarian cancer	Downregulated	SIRT6 inhibits Notch3 expression and cell proliferation.	[57]
Glioma	Downregulated	SIRT6 suppresses PCBP2 expression and cell proliferation.	[59]
		SIRT6 inhibits JAK2/STAT3 pathway and reduces cell survival.	[44]
Non-Small Cell Lung Cancer (NSCLC)	Downregulated	SIRT6 inhibits Twist-1 expression suppressing metastatization and EMT.	[62]
Pancreatic Ductal Adenocarcinoma (PDAC)	Downregulated	SIRT6 inhibits Lin28b expression and c-Myc recruitment suppressing cancer progression and metastatization.	[65]
Colorectal cancer (CRC)/Colon cancer	Downregulated	SIRT6-mediated deacetylation induces TRF2 ubiquitination, leading to proteasomal degradation	[21]
		USP10 inhibits SIRT6 degradation and blocks tumor growth via p53 and SIRT6-mediated degradation of c-Myc.	[53]
		miRNA-34c-5p inhibits SIRT6 expression and activates JAK2/STAT3 pathway inhibiting apoptosis.	[68]
Nasopharyngeal Carcinoma (NPC)	Downregulated	SIRT6 downregulates Bcl-2 and NF-κB and induces apoptosis mediated through upregulation of Bax and cleaved caspase-3.	[63]
Endometrial cancer	Downregulated	SIRT6 inhibits survivin expression promoting apoptosis	[70]
Melanoma	Downregulated	SIRT6 increases FoxO3a expression levels	[71]
		SIRT6 downregulates IGFBP2 through, thus impairing the activation of IGF-1R and AKT pathway, responsible of cancer cell survival and drug resistance.	[79]
		SIRT6-mediated suppression of IGF-AKT signaling stimulates autophagy, a tumor protective factor only at early stages.	[89]
Hepatocellular Carcinoma (HCC)	Downregulated	SIRT6 induces apoptosis via upregulation of cleaved caspase-3 and ERK1/2 pathway inhibition.	[61]
		SIRT6 deacetylates PKM2 inducing its nuclear export and blocks metastatization.	[64]
		SIRT6 inhibits survivin expression and induces apoptosis.	[39]
Hepatocellular Carcinoma (HCC)	Upregulated	SIRT6 downregulates Bax and blocks apoptosis.	[82]
		SIRT6 deacetylates Ku70 and blocks Bax-mediated apoptosis.	[94]
		SIRT6 promotes proliferation and invasion inducing ERK1/2 pathway and Bcl-2 expression and downregulating Bax and cleaved caspase-3.	[55]
		SIRT6 promotes proliferation and cell death evasion increasing p-AKT levels and XIAP expression.	[83]
		SIRT6 inhibits miR-125b expression and apoptosis.	[48]
		Reciprocal regulation between SIRT6 and miR-122 correlates with tumor progression.	[47]
Prostate cancer	Upregulated	SIRT6 knockdown increases sensitivity to chemotherapy, DNA damage, cell cycle arrest in G1, Bcl-2 downregulation and apoptosis induction.	[84]
Breast cancer	Upregulated	SIRT6 increases resistance to epirubicin and paclitaxel and negatively modulates the acetylation status and expression of FoxO3a and p53.	[85]
Osteosarcoma	Upregulated	SIRT6 facilitates DNA repair in cancer cells, leading to doxorubicin resistance. SIRT6 also inhibits the expression of Bax, cleaved-PARP1 and cleaved-caspase3 and increases Bcl-2 expression leading to apoptosis evasion.	[95]
Squamous Cell Carcinoma (SCC)	Upregulated	Under UVB radiation, AKT induces SIRT6 expression that promotes COX-2 action and AMP-activated protein kinase (AMPK) repression leading to cell survival and proliferation.	[86]
		SIRT6 is silenced by miR-34a and its downregulation induces cell differentiation and reduces cancer cell proliferation potential	[45]
Melanoma	Upregulated	SIRT6 activity increases the levels of the phosphatidylethanolamine-conjugated protein LC3-II, a crucial autophagosome initiator.	[87]
		SIRT6 suppresses IGF-AKT signaling, thus stimulating autophagy which is a protective factor at early stages, but promotes tumor development at later stages.	[89]
Diffuse Large B-Cell Lymphoma (DLBCL)	Upregulated	SIRT6 activates PI3K/AKT/mTOR pathway, thus promoting cancer proliferation.	[91]
		SIRT6 knockdown determines upregulation of the oncosuppressor FoxO1.	
Acute Myeloid Leukemia (AML)	Upregulated	SIRT6 increases cell death resistance via DNA-PKcs and CtIP deacetylation.	[92]
Multiple Myeloma (MM)	Upregulated	SIRT6 downregulates MAPK pathway genes suppressing cell proliferation.	[96]
		SIRT6 increases resistance to DNA-damaging agents through inhibition of ERK2/p90RSK signaling.	

**Table 3:** Most relevant SIRT6 activators.

Compound	Structure	Effect on SIRT6 Activity	Cellular and In Vivo Effects	Reference(s)
**3c** Cyanidin	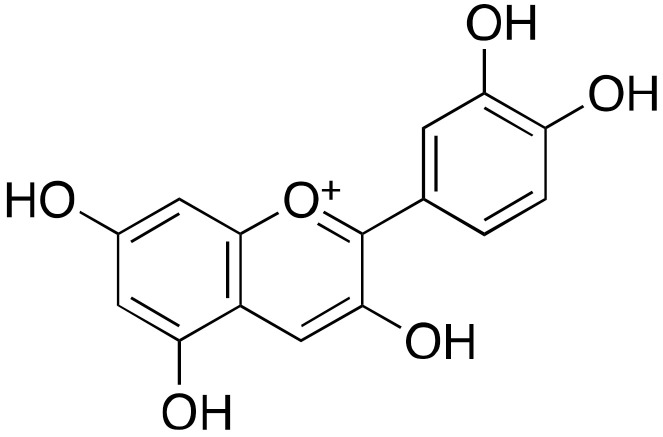	EC_50_ = 460 μM ×55 max activation (deacetylation)	In Caco-2 cells: dose-dependent SIRT6 upregulation; increased expression of FoxO3α. Decreased expression of Twist1 and GLUT1.	[98]
**4** UBCS039	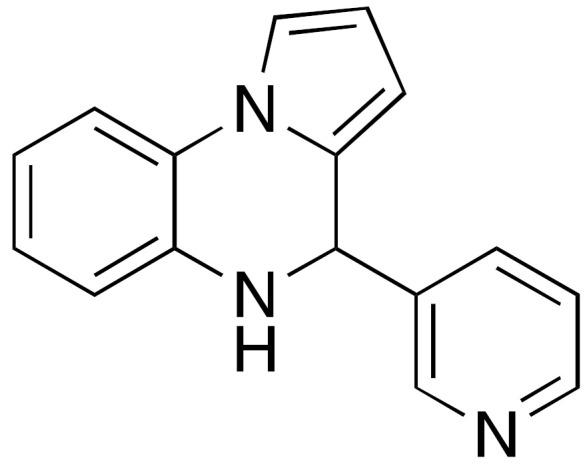	EC_50_ = 38 μM ×3.5 max activation (deacetylation)	SIRT6 activation in NSCLC, colon, epithelial cervix carcinoma, and fibrosarcoma. Decrease of H3K9 and H3K56 acetylation and autophagy-related cell death.	[99,100]
**5a** MDL-800	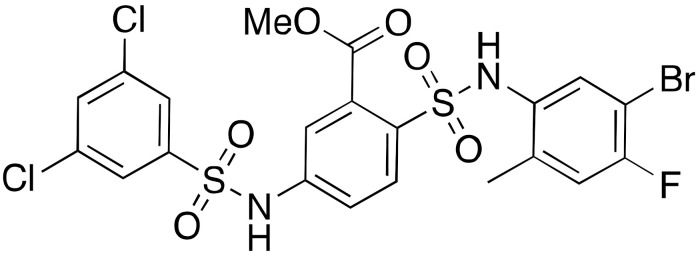	EC_50_ = 10.3 μM ×22 max activation (deacetylation)	Dose-dependent decrease of H3K9Ac and H3K56Ac in HCC and NSCLC causing cell cycle arrest. HCC tumor growth suppressed also in mouse xenograft models.	[101,102]
**5c** MDL-811	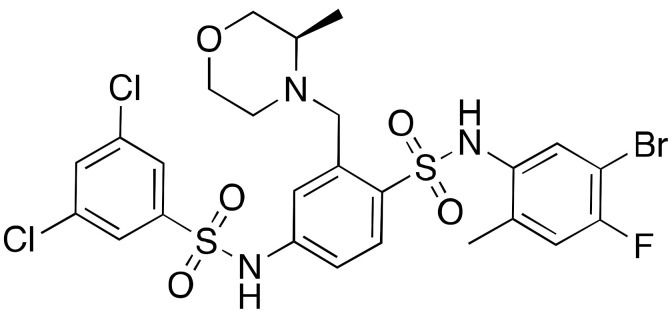	EC_50_ = 5.7 μM (deacetylation)	Dose-dependent reduction of H3K9Ac, H3K18Ac, and H3K56Ac levels in different CRC cell lines and antiproliferative effects associated with marked G0/G1 cell cycle arrest. CRC growth suppressed in patient-derived organoids and anti-tumor efficacy in cell line-derived and patient-derived xenografts.	[103]
**6**	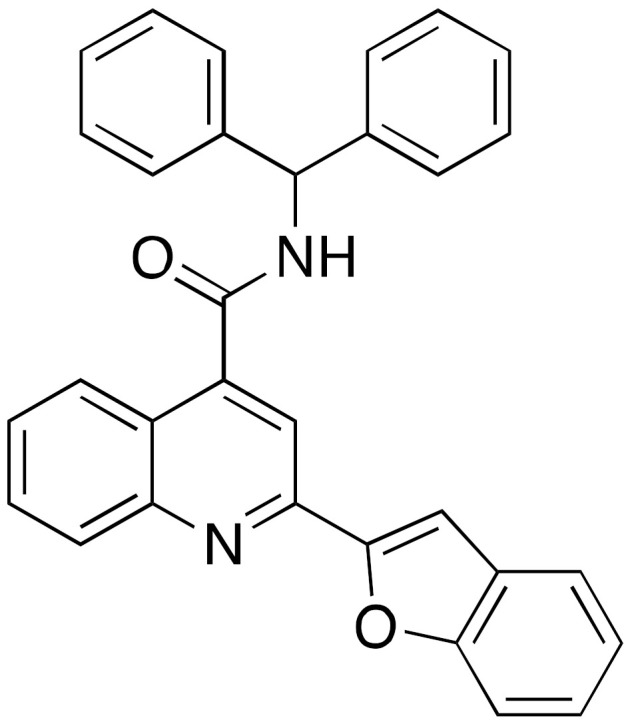	EC_50_ = 5.35 μM (deacetylation) EC_50_ = 8.91 μM (demyristoylation)	Suppression of PDAC cells proliferation cell cycle arrest in G2. Anti-tumor activity in a human pancreatic tumor xenograft mouse model associated with decrease of H3K9 acetylation levels.	[104]

**Table 4:** Most relevant SIRT6 inhibitors.

Compound	Structure	Effect on SIRT6 Activity	Cellular and In Vivo Effects	Reference(s)
**9b** BHJH-TM3	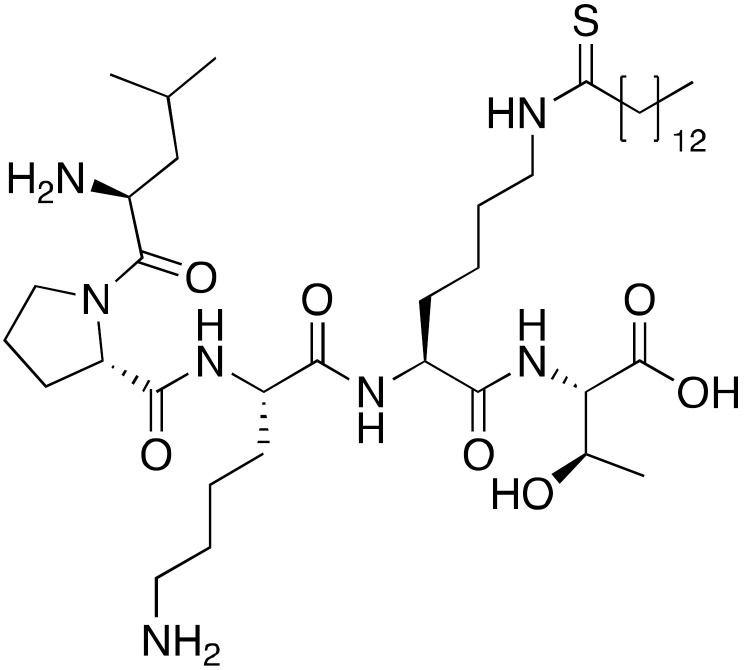	IC_50_ = 8.1 μM (demyristoylation)	SIRT6 inhibition and decreased TNF-α fatty acylation in HEK293T cells.	[114]
**11b** OSS_128167	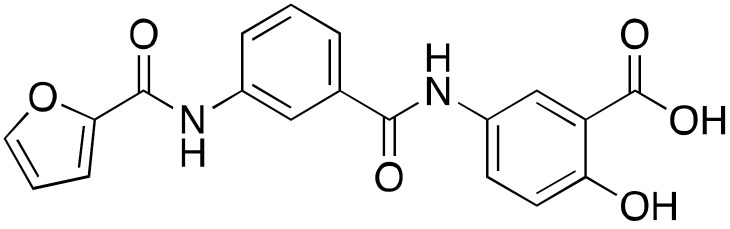	IC_50_ = 89 μM (deacetylation)	Augmented H3K9 acetylation and TNF-α secretion in BxPC3 cells. GLUT1 upregulation and consequent increased glucose uptake in L6 rat myoblasts and BxPC3 cells.	[115]
			Sensitization of MM cell lines to DNA-damaging agents.	[96]
			Suppression of DLBCL cell proliferation; induction of apoptosis and cell cycle arrest.	[91]
			Tumor growth reduction in DLBCL mouse xenograft.	
**12b**	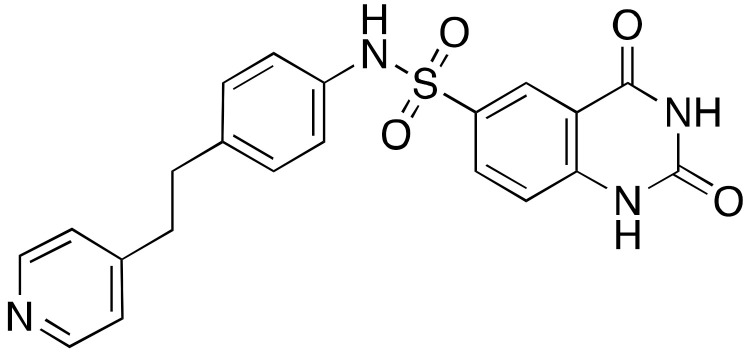	IC_50_ = 37 μM (deacetylation)	Increased H3K9 acetylation in BxPC3. Augmented glucose uptake in L6 rat myoblasts and BxPC3 cells. Sensitization of BxPC3 cells to gemcitabine. Enhancement of olaparib anticancer activity in Capan-1 cells.	[116]
**13b**	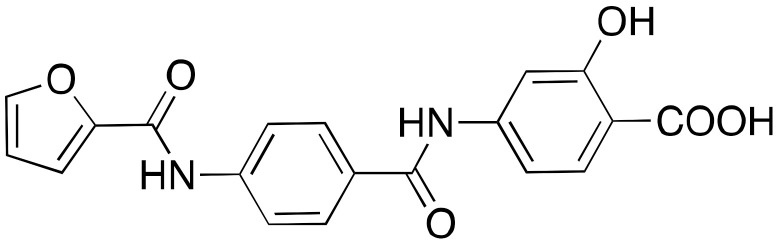	IC_50_ = 22 μM (deacetylation)	Increased H3K9 acetylation and glucose uptake in PBMCs. Impaired TNF-α secretion and T lymphocyte proliferation. Sensitization of pancreatic cancer cells to gemcitabine.	[117]
**14a** A127-(CONHPr)-B178	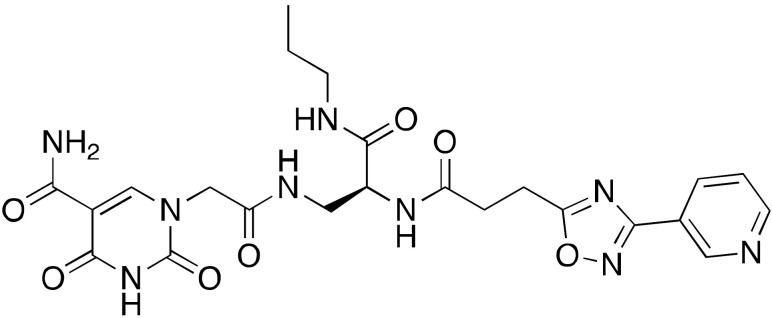	IC_50_ = 6.7 μM (demyristoylation)	Increase of DNA-damage markers and telomere-dysfunction induced foci in HUVECs. Reduction in TNF-α levels.	[118]
**15**	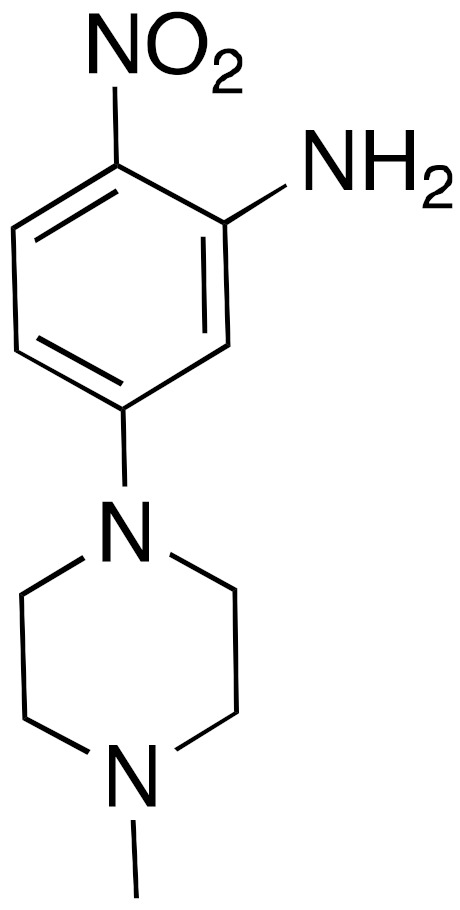	IC_50_ = 4.93 μM (deacetylation)	Dose-dependent increase of H3K9 and H3K18 acetylation levels in BxPC-3 cells. Increased GLUT-1 expression levels. Reduction of blood glucose content in a mouse model of type 2 diabetes.	[119]

## Abbreviations

ADP	Adenosine diphosphate
AML	Acute myeloid leukemia;
AMP	Adenosine monophosphate
AP-1	Activator protein 1
ATP	Adenosine triphosphate
AMPK	AMP-activated protein kinase
Bax	Bcl-2 associated X protein
BER	Base excision repair
cAMP	Cyclic adenosine monophosphate
CHIP	Carboxyl terminus of Hsp70-Interacting protein
CREB	cAMP response element-binding protein
DSB	Double strand break
DLBCL	Diffuse large B-cell lymphoma
EC_50_	Half maximal effective concentration
EMT	Epithelial-mesenchymal transition
FFA	Free fatty acid
HCC	Hepatocellular carcinoma
HDAC	Histone deacetylase
HIF- α	Hypoxia-inducible factor 1α
HP1 α	Heterochromatin protein 1α;
HR	Homologous recombination
IC_50_	Half maximal inhibitory concentration
IGF	Insulin-like growth factor
IGFBP2	Insulin-like growth factor-binding protein 2
IL-6	Interleukin-6
IL-8	Interleukin-8
IPSCs	Induced pluripotent stem cells
L1	Long interspersed element 1
MAPK	Mitogen-activated protein kinase
MDM2	Mouse double minute 2 homolog
miRNA	micro-RNA
MM	Multiple myeloma
NAD^+^	Nicotinamide adenine dinucleotide
NHEJ	Non-homologous end-joining
NPC	Nasopharyngeal carcinoma
NSCLC	Non-small cell lung cancer
PARP1	Poly [ADP-ribose] polymerase 1
PCPB2	Poly(C)-binding protein 2
PCD	Programmed cell death
PDAC	Pancreatic ductal adenocarcinoma
PKA	Protein kinase A
PKM2	Pyruvate kinase M2
ROS	Reactive oxygen species
SIRT	Sirtuin
SCC	Skin squamous cell carcinoma
SUMO	Small ubiquitin-related modifier
TNF- α	Tumor necrosis factor α
TRF2	Telomere repeat binding factor 2
UTR	Untranslated region
XIAP	X-linked inhibitor of apoptosis protein
